# Quality of life-related and non-quality of life-related issues in ICU survivors and non-ICU-treated controls: a multi-group exploratory factor analysis

**DOI:** 10.1186/s13054-024-04890-7

**Published:** 2024-03-29

**Authors:** Johan Malmgren, Stefan Lundin, Ann-Charlotte Waldenström, Christian Rylander, Elias Johannesson

**Affiliations:** 1 Department of Anaesthesiology and Intensive Care Medicine, Institute of Clinical Sciences, Sahlgrenska Academy, Sahlgrenska University Hospital, University of Gothenburg, Blå Stråket 5, 413 45 Gothenburg, Sweden; 2 Department of Oncology, Institute of Clinical Sciences, Sahlgrenska Academy, Sahlgrenska University Hospital, University of Gothenburg, 413 45 Gothenburg, Sweden; 3 Anesthesiology and Intensive Care, Department of Surgical Sciences, Uppsala University, Uppsala, Sweden; 4 Department of Social and Behavioural Studies, University West, Trollhättan, Sweden

**Keywords:** Critical care, Intensive care unit, Critical illness, Quality of life, Long-term adverse effects, Questionnaire, Patient-reported outcome, Survivorship

## Abstract

**Background:**

Quality of life (QoL) is a key outcome measure in healthcare. However, the heterogeneity in its definitions presents challenges in the objective evaluation of improvement. Universal questionnaires, tailored for a broad demographic group, inadequately represent the unique experiences of intensive care unit (ICU) survivors, including a lack of ability to discriminate issues related to QoL from issues that do not.

**Methods:**

Using a 218-item, 13-domain provisional questionnaire, we assessed 395 adult ICU survivors, with a minimum 72-h stay at one of three Swedish university hospital ICUs, at 6 months to three years post-discharge. Their responses were compared to those of 195 controls, matched for age and sex and randomly recruited from the Swedish Population Registry. By multi-group exploratory factor analysis, we compared dimensionality in QoL perceptions between the two groups, emphasising patterns of correlation to 13 domain-specific QoL questions. Model fit was assessed using information criteria. Internal consistency reliability for each scale was determined using McDonald’s omega or Cronbach’s alpha. All analyses were conducted using Mplus, applying full information maximum likelihood to handle missing data.

**Results:**

All domains except Cognition had a subset of questions correlating to the domain-specific QoL question in at least the ICU survivor group. The similarity between the two groups varied, with Physical health, Sexual health and Gastrointestinal (GI) functions mainly correlating the same issues to QoL in the two groups. In contrast, Fatigue, Pain, Mental health, activities of daily living, Sleep, Sensory functions and Work life showed considerable differences. In all, about one-fourth of the issues correlated to QoL in the ICU survivor group and about one-tenth of the issues in the control group.

**Conclusions:**

We found most issues experienced by ICU survivors to be unrelated to quality of life. Our findings indicate that the consequences of post-ICU issues may play a more significant role in affecting QoL than the issues themselves; issues restricting and affecting social life and work life were more related to QoL in ICU survivors than in non-ICU-treated controls. Caution is advised before associating all post-ICU problems with an effect on quality of life.

*Trial registration*: ClinicalTrials.gov Ref# NCT02767180; Registered 28 April 2016.

**Supplementary Information:**

The online version contains supplementary material available at 10.1186/s13054-024-04890-7.

## Introduction

With multiple frameworks for the definition of quality of life (QoL) having been proposed, health-related quality of life (HRQoL) is perhaps the most commonly described and used concept within healthcare [1–4]. Differently defined by the various frameworks, the relationship between objective and subjective indicators, their mutual relevance, and even whether there is a distinction between them is contested [5–7]. With diverse definitions of QoL, difficulties for clinicians to objectively assess patients' QoL are common and add a need for comprehensive questionnaires as measurement proxies for QoL. Improvements in survival are increasingly challenging to demonstrate in trials, and intensive care is no exception to these needs and problems; the generic HRQoL questionnaires SF-36 and EQ-5D are commonly used but are both developed for a general population [8]. Thus, their dismal results in measuring ICU survivors' views on important issues are not surprising [9, 10].

We have previously reported on the development and initial evaluation of a novel questionnaire for measuring burdens after intensive care [11]. Compared to a non-ICU-treated control group, ICU survivors reported a worse current state in almost 80% of the issues tested for. However, while these issues affect daily life, not all necessarily affect QoL. This study aims to compare patterns in how ICU survivors and non-ICU-treated controls perceive issues across different areas specifically in relation to QoL.

## Methods

### Study setting and populations

The development of the provisional questionnaire has been reported elsewhere [11]. In short, thirty-two adult ICU survivors were interviewed at least 6 months after ICU discharge. One hundred ninety-five unique issues were extracted from the interviews. These issues were rephrased into questions, categorised into 13 domains (Cognition, Fatigue, Physical health, Pain, Mental health, Activities of Daily Living [ADL], Sleep, Appetite and Alcohol, Sexual health, Sensory functions, Gastrointestinal functions, Urinary tract functions, and Work life), and converted into a questionnaire. Each domain ended with a summarising QoL question, i.e. "*To what extent has difficulties with [domain] affected your quality of life for the past month?"*.

The questionnaire was used in a cross-sectional study comparing responses between ICU survivors and a non-ICU-treated control group. Eligible participants were all adult ICU survivors admitted between February 2013 and December 2015 to one of three mixed ICUs at a university hospital in Gothenburg, Sweden, with a minimum ICU length of stay of 72 h. Patients with neurological/neurosurgical admission diagnoses were excluded. The evaluation was performed 6 months to three years after ICU discharge. A non-ICU-treated control group, matched by age and sex, was randomly recruited from the Swedish Population Registry. An additional question regarding previous ICU care was added to the questionnaire for the control group and constituted an exclusion criterion.

### Statistical analysis

A multi-group exploratory factor analysis (MG-EFA) was performed to examine the factor structure of each domain. MG-EFA is a technique to compare dimensionality across a grouping variable, thus allowing for a comparison between the ICU survivor group and the non-ICU-treated control group simultaneously [12]. As such, MG-EFA is suitable for studying QoL perceptions because of its multidimensional nature, and the analysis may bring insight into differences and similarities between two groups [13]. Similarly to regular exploratory factor analysis, all items are allowed to load on all factors within their domain. Given that MG-EFA evaluates both groups simultaneously, results should be understood in the context of comparing one group to the other, rather than considering them individually. Therefore, observed patterns might differ if each group had been analysed on its own. Importantly, all issues loading strongly in the same dimension as the domain-specific QoL question will be interpreted as correlating to QoL, while issues loading strongly in other dimensions will be interpreted as significant issues in daily life but not related to QoL. Theoretically, the domain-specific QoL question can load strongly in all dimensions, thus indicating that all issues in a domain are QoL-correlated.

To evaluate which model was best represented by our data (optimal model fit) we used Akaike information criterion (AIC) and Bayesian information criterion (BIC). AIC and BIC indicate whether adding one more dimension to the current model is better than the previous model with one dimension less fitted. A decrease in these statistical indicators is associated with an improvement in fit and model selection, and the lowest AIC/BIC is therefore the best trade-off between model fit and model complexity [14]. BIC is frequently used when models are compared and reported to perform better than AIC [15]. Therefore, we will report both these indicators for model selection but use BIC as the primary criterion for determining the number of dimensions for each domain across both groups.

Domains not converging for any factor solutions or exhibited lower BIC values were excluded from further comparison.

The internal consistency reliability of each scale was measured for both groups using McDonald's omega for domains with at least three factor loadings and Cronbach's alpha for domains with less than three factor loadings. Omega is a more robust index than Cronbach's alpha when estimating the reliability of questionnaires but requires at least three factor loadings [16]. Reliability estimates were graded accordingly, with a low statistic suggesting item reduction in the questionnaire [17, 18].

All analyses were performed with Mplus 8.0 (Muthén & Muthén, 2017) with 100,000 iterations for factor solutions. Mplus applies full information maximum likelihood (FIML) to missing data by default. FIML has been demonstrated to generate accurate and unbiased estimates even when the normality assumption is violated if the missing mechanism is missing completely at random or missing at random [19, 20].

Two gender-specific questions were removed for this analysis due to analytical reasons (*Vaginal dryness* and *Erectile dysfunction*).

## Results

**Fig. 1:**
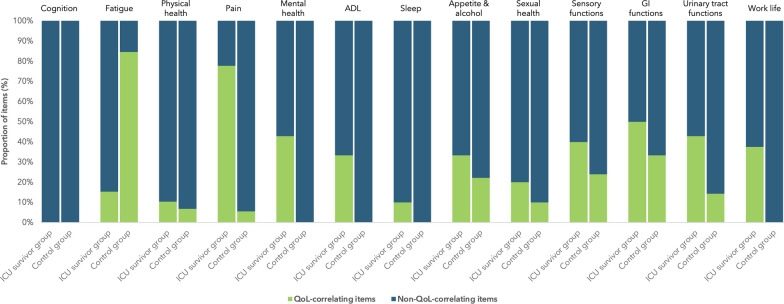
Proportion of items affecting quality of life. Bar graph showing the proportion of items affecting quality of life versus items not affecting quality of life in the ICU survivor group compared to the control group. All investigated domains are shown, including those where there was no correlation to quality of life at all (e.g. cognition)

Demographics for both groups are shown in Table 1. The initial test for model fit showed that all domains converged for a factor solution, thus excluding no domain for further analysis. All domains except Cognition had a subset of items correlating to the domain-specific QoL question in at least the ICU survivor group. Factor loadings and reliability coefficients for all questions are shown in Table 2 for both groups. The proportion of items affecting QoL versus those not affecting QoL is shown in Fig. 1. Fatigue.

**Table 1:** Demographics and characteristics for ICU survivors and control group

	ICU survivors (* n* = 395)	Controls (* n* = 195)	*p*-value	Total (*N*)
Age, years; median (IQR)	65.0 (18)	65.0 (15)	0.56	589
Body mass index; median (IQR)	26.0 (7)	25.4 (5)	0.17	555
Smoker; n (%*)	15 (13)	15 (11)	0.01	109
Male; n (%*)	239 (61)	117 (60)		
Median SAPS score (range)	59 (16–100)	N/A		
Days of ICU length of stay; median (range)	5.6 (3.0–78.6)	N/A		
Mechanical ventilation; %	78	N/A		
Days of mechanical ventilation; median (range)	4.0 (0–74)	N/A		

*Percent of responding participants

**Table 2:** MG-EFA correlation matrix comparing ICU survivor group to non-ICU-treated control group

		ICU survivor group				Non-ICU-treated control group			
	Cognition	Dim 1	Dim 2	Dim 3		Dim 1	Dim 2	Dim 3	
COG1	Difficulties finding words	**0.79**	0.01	0.00		**0.44**	0.25	0.08	
COG2	Difficulties finishing sentences	**0.87**	0.01	0.02		**0.65**	0.14	− 0.01	
COG3	Losing the thread easily	**0.70**	0.16	0.06		**0.75**	0.03	− 0.01	
COG4	Don't remember what you have said	0.10	**0.68**	0.04		**0.78**	0.00	− 0.07	
COG5	Don't remember what you have done	− 0.13	**0.88**	0.02		**0.83**	− 0.05	− 0.22	
COG6	Think you have done something but you haven't	0.17	**0.69**	− 0.03		**0.64**	0.11	− 0.15	
COG7	Forgotten what you were going to get	0.10	**0.32**	**0.36**		0.21	**0.45**	− 0.04	
COG8	Need to be reminded to do an activity	0.03	**0.52**	**0.33**		0.25	**0.38**	0.12	
COG9	Difficulties thinking clearly	**0.41**	− 0.02	**0.51**		**0.46**	**0.34**	0.11	
COG10	Need for memos	0.10	0.16	**0.40**		0.03	**0.67**	− 0.18	
COG11	Difficulties remembering names	**0.34**	0.08	**0.28**		− **0.27**	**0.84**	0.01	
COG12	Difficulties remembering general knowledge	0.18	0.22	**0.43**		0.17	**0.64**	− 0.08	
COG13	Difficulties remembering what you have read	0.24	0.02	**0.59**		0.05	**0.80**	− 0.02	
COG14	Difficulties remembering previous TV-episode	0.03	**0.45**	**0.36**		0.04	**0.48**	**0.31**	
COG15	Difficulties learning new things	0.18	0.11	**0.58**		− 0.03	**0.60**	**0.45**	
COG16	Difficulties remembering numbers	0.03	0.20	**0.53**		− 0.12	**0.80**	0.01	
COG17	Difficulties being on time	0.08	**0.30**	**0.35**		**0.66**	− 0.02	0.02	
COG18	Missed a scheduled meeting	0.02	**0.39**	0.23		0.09	0.17	− 0.23	
COG19	Mistaken which day of the week	− 0.04	**0.41**	**0.37**		**0.60**	0.02	0.22	
COG20	Forgotten where you have put something	− 0.02	**0.39**	**0.40**		− 0.02	**0.70**	− 0.10	
COG21	Need to double-check things	− 0.02	0.13	**0.48**		0.04	**0.51**	0.02	
COG22	Difficulties finding your way around	− 0.01	0.22	**0.38**		0.16	**0.33**	**0.26**	
COG23	Someone has said that you have memory problems	− 0.13	**0.52**	**0.39**		**0.43**	0.19	− 0.03	
COG24	Worrying about having memory problems	0.19	**0.29**	**0.39**		**0.50**	0.00	**0.56**	
COG25	Difficulties taking initiatives	0.01	− **0.34**	**0.91**		**0.34**	0.13	**0.46**	
COG26	Difficulties prioritizing	− 0.05	− **0.30**	**0.86**		**0.29**	**0.40**	0.19	
COG27	Difficulties concentrating	**0.24**	− 0.21	**0.79**		**0.31**	**0.44**	0.17	
COG28	Difficulties finding alternative solutions	− 0.08	0.03	**0.75**		**0.53**	0.13	**0.30**	
COG29	Time spent reading	0.12	0.00	− 0.22		− 0.09	− 0.06	0.02	
COG30	*Memory/thinking difficulties affecting QoL*	**0.37**	0.04	**0.49**		**0.39**	0.21	**0.43**	
COG31	Worrying about your memory/thinking	**0.29**	− 0.01	**0.49**		**0.48**	− 0.02	**0.67**	
	Reliability	0.88	0.84	0.90		0.85	0.87	–*	

	Fatigue	Dim 1	Dim 2			Dim 1	Dim 2		
FAT1	Need for daytime rest	0.23	**0.53**			***0.65***	0.08		
FAT2	Tough getting started doing things	**0.30**	**0.51**			***0.68***	− 0.08		
FAT3	Difficulties finishing things due to feeling exhausted	**0.32**	**0.54**			***0.85***	− 0.01		
FAT4	Difficulties doing things under pressure of time	**0.56**	**0.34**			***0.82***	0.08		
FAT5	Difficulties multitasking due to feeling exhausted	**0.48**	**0.34**			***0.91***	0.08		
FAT6	Tired from reading	**0.86**	− 0.02			***0.81***	0.12		
FAT7	Tired from watching TV	**0.79**	0.00			***0.80***	0.00		
FAT8	Tired from conversation between more than two people	**0.56**	**0.26**			***0.85***	− 0.03		
FAT9	Fallen asleep when reading	**0.38**	0.07			**0.45**	**0.35**		
FAT10	Fallen asleep during a conversation	**0.28**	− 0.01			**0.52**	0.18		
FAT11	Tiredness affecting work	− 0.03	***0.90***			***0.88***	0.14		
FAT12	Tiredness limiting social activities	− 0.01	***0.86***			***0.78***	− 0.20		
FAT13	*Tiredness affecting QoL*	0.04	***0.88***			***0.92***	− **0.37**		
FAT14	Worrying about feeling tired	0.30	**0.56**			***0.80***	− 0.19		
	Reliability	0.84*	0.92			0.96	–*		

	Physical health	Dim 1	Dim 2	Dim 3	Dim 4	Dim 1	Dim 2	Dim 3	Dim 4
PHYS1	Physical health in general	0.01	0.00	***0.79***	0.00	0.06	0.02	***0.74***	− 0.10
PHYS2	Reduced feeling in your face	0.29	0.12	0.05	**0.34**	**0.41**	**0.27**	− 0.04	0.23
PHYS3	Arm weakness	**0.52**	− 0.10	**0.42**	0.16	**0.36**	**0.26**	0.23	0.03
PHYS4	Reduced feeling in arms	**0.67**	0.03	0.03	**0.33**	**0.93**	0.03	− 0.12	− 0.03
PHYS5	Reduced feeling in hands/fingers	**0.69**	0.11	− 0.03	**0.26**	**0.69**	0.20	− 0.06	0.01
PHYS6	Raynaud's in fingers	**0.44**	0.21	0.06	**0.32**	**0.36**	0.23	0.08	− 0.09
PHYS7	Difficulties extending your wrist	**0.57**	0.00	− 0.02	0.20	0.17	0.13	− 0.16	0.21
PHYS8	Difficulties lifting/carrying lightweight objects	**0.88**	0.03	− 0.04	− 0.02	0.08	**0.86**	0.03	− 0.03
PHYS9	Difficulties turning on taps/opening jars	**0.86**	− 0.07	0.04	− 0.06	− 0.08	**0.90**	0.02	0.03
PHYS10	Difficulties using your hands	**0.80**	0.05	0.02	− 0.08	**0.25**	**0.27**	0.02	0.19
PHYS11	Leg weakness	0.12	**0.49**	**0.33**	− 0.05	0.05	0.19	**0.46**	**0.29**
PHYS12	Reduced feeling in legs	0.13	**0.83**	− 0.14	0.08	**0.76**	− 0.16	0.09	0.06
PHYS13	Reduced feeling in feet/toes	− 0.07	**0.90**	− 0.08	0.08	**0.67**	− 0.23	0.06	0.03
PHYS14	Restless legs	0.01	**0.33**	**0.23**	**0.27**	**0.31**	0.05	**0.26**	− 0.08
PHYS15	Dizziness when standing up	0.13	0.12	**0.33**	0.07	0.13	0.17	0.15	0.23
PHYS16	Losing balance easily	**0.30**	**0.34**	**0.24**	− 0.15	0.13	0.02	0.12	**0.65**
PHYS17	Difficulties climbing stairs	0.04	**0.43**	**0.40**	− **0.27**	− 0.13	0.02	**0.48**	**0.61**
PHYS18	Unsteady gait	**0.26**	**0.63**	0.04	− 0.23	0.13	− 0.01	0.03	**0.78**
PHYS19	Legs feeling heavy	− 0.02	**0.63**	0.17	− 0.06	0.10	0.02	0.14	**0.57**
PHYS20	Swollen legs/ankles	− 0.11	**0.46**	0.13	0.04	0.12	− 0.08	**0.26**	0.10
PHYS21	Raynaud's in toes	− 0.12	**0.73**	0.11	0.13	**0.31**	− 0.07	0.22	− 0.03
PHYS22	Foot drop	0.09	**0.67**	− 0.20	− 0.03	0.04	0.00	− 0.18	**0.72**
PHYS23	Contractures	0.16	**0.40**	0.23	− 0.04	− 0.01	0.21	**0.36**	**0.30**
PHYS24	Periods of heavy sweating	0.14	0.01	**0.32**	**0.38**	0.02	0.08	0.23	0.18
PHYS25	Able to walk six minutes	0.09	0.18	**0.24**	− **0.44**	− 0.18	− 0.12	− 0.04	**0.70**
PHYS26	Walking longer than 1 km	− 0.05	0.18	**0.36**	− **0.43**	− 0.04	− 0.10	0.21	**0.36**
PHYS27	Shortness of breath limiting your physical activities	− 0.01	− 0.02	***0.75***	− 0.17	− 0.03	− 0.04	**0.57**	0.14
PHYS28	Physically active ≥ 30 min	− 0.02	0.01	− **0.50**	**0.29**	0.01	0.08	− **0.42**	− 0.01
PHYS29	*Physical health affecting QoL*	0.00	0.13	***0.76***	0.03	− 0.01	0.01	***0.86***	0.01
PHYS30	Worrying about physical health	0.03	0.09	***0.72***	0.08	**0.16**	− 0.01	***0.80***	0.02
	Reliability	0.89	0.87	0.85	–*	0.82	0.85*	0.89	0.87

	Pain	Dim 1	Dim 2	Dim 3	Dim 4	Dim 1	Dim 2	Dim 3	Dim 4
PAIN1	Headaches	**0.26**	**0.29**	0.02	− 0.14	0.18	**0.26**	**0.24**	− 0.10
PAIN2	Finding normal touch bothersome	**0.27**	**0.28**	0.07	0.15	− 0.03	0.14	**0.38**	− 0.19
PAIN3	General body pain	**0.34**	**0.48**	0.03	− 0.05	0.19	**0.52**	0.19	0.07
PAIN4	Shoulder pain	**0.86**	0.00	− 0.03	− 0.21	**0.49**	− 0.07	**0.58**	0.01
PAIN5	Arm pain	**0.81**	− 0.03	0.08	0.09	**0.57**	0.07	**0.43**	0.05
PAIN6	Hand pain	**0.37**	0.23	0.08	**0.28**	**0.47**	**0.36**	− 0.04	− 0.03
PAIN7	Back pain	0.14	**0.55**	0.00	− 0.14	0.06	**0.31**	0.02	**0.33**
PAIN8	Chest pain	**0.43**	0.26	− 0.05	− 0.01	0.00	**0.34**	0.02	− 0.08
PAIN9	Abdominal pain	0.10	**0.34**	0.04	− 0.07	− 0.07	0.18	**0.30**	− 0.03
PAIN10	Leg pain	0.06	***0.64***	− 0.01	**0.39**	− 0.16	**0.68**	0.02	0.02
PAIN11	Foot pain	− 0.05	***0.61***	− 0.02	**0.41**	− 0.23	**0.35**	0.17	0.00
PAIN12	Use of painkillers	0.10	0.07	**0.46**	− **0.32**	− 0.05	**0.31**	0.08	0.22
PAIN13	Pain stopping planned activity	0.05	***0.66***	0.12	0.00	− 0.06	0.11	0.14	***0.68***
PAIN14	Painkillers to manage ADL	− 0.04	0.08	**0.79**	− 0.21	0.02	**0.41**	0.22	0.27
PAIN15	Painkillers for sufficient sleep	− 0.01	− **0.36**	**0.91**	− 0.01	− 0.08	0.05	**0.79**	− 0.01
PAIN16	Pain makes going to sleep difficult	0.08	0.03	**0.75**	0.18	0.10	0.08	**0.67**	0.09
PAIN17	Woken by pain	0.15	**0.24**	**0.46**	0.10	− 0.01	− 0.08	**0.52**	**0.47**
PAIN18	*Pain affecting QoL*	− 0.04	**0.78**	0.15	− 0.03	0.08	**0.34**	− 0.01	***0.69***
PAIN19	Worrying about pain	− 0.05	***0.76***	0.11	0.03	0.00	**0.67**	− 0.02	**0.29**
	Reliability	0.81	0.85	0.87	–*	–*	0.69*	0.75*	0.82*

	Mental health	Dim 1	Dim 2	Dim 3		Dim1	Dim 2	Dim3	
PSYCH1	Crying easily	**0.27**	**0.25**	0.18		**0.48**	0.06	0.03	
PSYCH2	Feeling short-tempered	**0.92**	− 0.01	0.06		**0.98**	0.00	**0.32**	
PSYCH3	Losing patience easily	**0.88**	0.04	0.03		**0.95**	− 0.01	**0.26**	
PSYCH4	Difficulties feeling warmth towards family members	**0.41**	**0.29**	− 0.05		**0.43**	0.19	− 0.11	
PSYCH5	Difficulties unwinding	**0.23**	**0.51**	− 0.03		**0.50**	**0.36**	0.01	
PSYCH6	Worrying about little things	**0.26**	**0.59**	− 0.02		**0.67**	0.18	0.05	
PSYCH7	Feeling low-spirited	**0.15**	***0.77***	− 0.06		**0.57**	0.39	− 0.01	
PSYCH8	Feeling depressed	0.00	***0.89***	− 0.07		**0.52**	**0.40**	− 0.01	
PSYCH9	Periods of anxiety	− 0.01	***0.86***	**0.15**		**0.18**	**0.67**	0.00	
PSYCH10	Panic attacks	0.03	**0.57**	**0.26**		− 0.19	**0.70**	0.02	
PSYCH11	Feelings of hopelessness	0.04	***0.80***	0.02		− 0.01	**0.83**	− 0.04	
PSYCH12	Feelings of life being meaningless	0.08	***0.76***	− 0.01		0.17	**0.71**	0.05	
PSYCH13	Cannot stop worrying about being ill	0.01	***0.60***	0.03		**0.29**	0.25	− 0.08	
PSYCH14	Low self-confidence	− 0.06	***0.80***	− 0.01		**0.33**	**0.45**	− 0.02	
PSYCH15	Low self-esteem	− 0.04	***0.83***	0.05		**0.48**	0.39	− 0.01	
PSYCH16	Able to laugh at things	0.00	− **0.50**	**0.61**		**0.00**	0.20	**0.87**	
PSYCH17	Able to look forward to things	0.00	− **0.57**	**0.65**		− **0.19**	0.00	**0.72**	
PSYCH18	Difficulties talking about your illness to family/close friends	0.03	**0.49**	0.06		**0.17**	0.11	− **0.29**	
PSYCH19	Feeling that others think you talk too much about your illness	**0.28**	**0.25**	0.18		**0.32**	0.01	0.05	
PSYCH20	Counselling (pre-ICU vs "previously")	− 0.12	**0.39**	0.08		**0.32**	0.05	− 0.03	
PSYCH21	*Mental health affecting QoL*	0.07	***0.72***	0.04		**0.55**	0.18	− 0.11	
PSYCH22	Worrying about psychological/mental health	− 0.05	***0.84***	0.09		**0.35**	0.34	− 0.13	
	Reliability	0.91*	0.94	0.83*		0.85	0.85	0.79*	

	ADL	Dim 1	Dim 2			Dim 1	Dim 2		
ADL1	Home care	**0.55**	0.22			0.14	**0.96**		
ADL2	Personal assistant	**0.46**	**0.28**			**0.97**	0.16		
ADL3	Help showering	**0.79**	**0.41**			**0.83**	**0.52**		
ADL4	Help getting dressed	**0.90**	**0.28**			**0.88**	**0.66**		
ADL5	Help moving between chair and bed	**0.89**	**0.32**			**0.99**	**0.29**		
ADL6	Support sitting up	0.10	**0.49**			**0.99**	**0.33**		
ADL7	Help visiting the toilet	**0.88**	0.15			**0.97**	0.16		
ADL8	Help with shopping	**0.51**	***0.65***			**0.60**	**0.92**		
ADL9	Help with cooking	**0.54**	***0.62***			**0.63**	**0.89**		
ADL10	Help with housework	**0.52**	***0.65***			**0.64**	**0.83**		
ADL11	Help with medication	**0.41**	**0.44**			0.12	**0.85**		
ADL12	Avoided travelling in a car	0.09	**0.25**			0.10	**0.26**		
ADL13	Avoided taking public transport	0.10	***0.60***			**0.83**	**0.33**		
ADL14	Help managing bills	**0.41**	**0.49**			0.10	**0.95**		
ADL15	*Daily activities affecting QoL*	**0.33**	***0.88***			**0.53**	**0.55**		
ADL16	Worrying about daily activities	0.18	***0.85***			**0.43**	**0.79**		
	Reliability	0.92	0.85			0.96	0.94		

	Sleep	Dim 1	Dim 2	Dim 3		Dim 1	Dim 2	Dim 3	
SLEEP1	Need for daytime nap	0.05	0.09	0.22		**0.92**	0.00	− 0.40	
SLEEP2	Difficulties going to sleep	0.16	0.00	**0.58**		− 0.02	**0.31**	**0.43**	
SLEEP3	Need for sleeping pills	− 0.15	0.27	0.25		0.00	− 0.31	**0.72**	
SLEEP4	Anxiety before going to sleep	0.12	**0.60**	0.12		− 0.06	0.07	**0.53**	
SLEEP5	Difficulties going back to sleep	**0.36**	− 0.02	**0.44**		− 0.01	**0.77**	0.01	
SLEEP6	Night-time worrying	0.20	0.04	0.00		0.16	**0.71**	0.00	
SLEEP7	Nightmares	0.00	**0.92**	− 0.18		**0.37**	0.18	0.04	
SLEEP8	Nightly sweats disturbing sleep	0.04	**0.37**	0.12		**0.46**	0.19	0.00	
SLEEP9	Heart palpitations disturbing sleep	− 0.02	**0.59**	0.00		**0.44**	− 0.24	0.23	
SLEEP10	*Sleep problems affecting QoL*	0.04	0.03	***0.77***		0.07	**0.39**	**0.47**	
SLEEP11	Worrying about sleep	− 0.02	0.14	***0.71***		**0.30**	0.01	**0.66**	
	Reliability	–*	0.73*	0.81*		–*	0.79*	0.63*	

	Appetite & Alcohol	Dim 1	Dim 2	Dim 3		Dim 1	Dim 2	Dim 3	
A&A1	Bothersome thirst	**0.29**	− 0.11	0.10		**0.57**	− 0.03	0.03	
A&A2	Difficulties chewing	**0.55**	− 0.01	0.05		**0.24**	**0.27**	0.14	
A&A3	Sugar cravings	0.10	− **0.27**	**0.34**		0.03	**0.24**	− 0.07	
A&A4	Poor appetite	***0.80***	0.09	− 0.02		**0.97**	0.01	− 0.03	
A&A5	Alcohol, how often	− 0.02	**0.66**	− 0.13		− 0.20	− 0.03	**0.25**	
A&A6	Alcohol, how many glasses on a typical day	0.02	**0.76**	0.10		− 0.14	0.02	**0.34**	
A&A7	Alcohol, how often 6 or more glasses	**0.15**	**0.81**	0.01		− 0.03	0.00	**0.28**	
A&A8	*Appetite affecting QoL*	***0.89***	− 0.02	0.06		**0.29**	***0.77***	0.01	
A&A9	Worrying about your appetite	***0.78***	0.02	− 0.04		− 0.01	***1.00***	− 0.06	
A&A10	*Alcohol affecting QoL*	0.00	0.01	***0.94***		0.01	**0.41**	***0.71***	
A&A11	Worrying about alcohol	− 0.01	0.16	***0.71***		− 0.01	− 0.01	***0.90***	
	Reliability	0.86	0.75	0.84*		–*	0.95*	0.85*	

	Sexual health	Dim 1	Dim 2			Dim 1	Dim 2		
SEX1	Difficulties handling physical closeness from loved ones	− 0.10	**0.24**			− **0.17**	**0.23**		
SEX2	Sex drive	**0.74**	**0.20**			**0.70**	0.07		
SEX3	Sexual activity	**0.89**	0.09			**0.91**	0.05		
SEX4	Sex life	**0.69**	**0.29**			**0.74**	**0.17**		
SEX5	Orgasm	**0.87**	0.00			**0.88**	0.00		
SEX6	Bothered by being naked in front of partner **	0.05	**0.45**			− 0.03	**0.36**		
SEX7	Surgical scars affecting sex life	0.09	***0.60***			− 0.03	**0.31**		
SEX8	Lack of energy affecting sex life	− **0.15**	**0.36**			0.04	**0.50**		
SEX9	Pain during sex	− 0.02	**0.30**			− **0.17**	0.11		
SEX10	*Problems with sex life affecting QoL*	− **0.17**	***0.73***			− 0.04	***0.87***		
SEX11	Worrying about sex life	0.00	***0.63***			0.12	***0.78***		
	Reliability	0.90	0.67			0.90	0.82*		

	Sensory functions	Dim 1	Dim 2	Dim 3		Dim 1	Dim 2	Dim 3	
SENS1	Reduced taste	***0.62***	0.10	0.25		0.08	**0.86**	0.05	
SENS2	Reduced smell	***0.62***	− 0.08	0.19		0.00	**0.75**	0.07	
SENS3	Reduced eyesight/vision	− **0.38**	0.04	− 0.07		− 0.16	− 0.03	− 0.07	
SENS4	Visual field	0.26	− 0.07	0.07		− 0.10	− 0.07	− 0.08	
SENS5	Colour vision	**0.42**	− 0.22	**0.30**		0.04	0.03	0.01	
SENS6	Sensitive to bright light	**0.44**	− 0.14	**0.28**		0.26	0.22	− 0.16	
SENS7	Blurred vision	**0.29**	− 0.02	− 0.12		0.16	0.08	− 0.02	
SENS8	Reduced hearing	− **0.38**	**0.78**	0.15		− 0.21	− 0.04	− 0.05	
SENS9	Sound hypersensitivity	**0.34**	− **0.35**	**0.33**		0.14	− 0.16	0.08	
SENS10	Poor hearing	**0.28**	− **0.66**	− **0.40**		**0.30**	0.07	− 0.10	
SENS11	Bothered by surrounding sounds	**0.57**	− **0.37**	**0.37**		***0.72***	0.01	0.13	
SENS12	Difficulties hearing what people say	**0.55**	− **0.61**	− **0.35**		***0.71***	**0.27**	0.18	
SENS13	Reduced hearing limiting social life	***0.60***	− **0.36**	− **0.39**		***0.62***	0.22	0.07	
SENS14	Sound hypersensitivity limiting social life	**0.54**	− 0.21	0.24		***0.74***	0.22	0.04	
SENS15	Tinnitus	0.21	− **0.28**	0.03		***0.79***	0.04	0.12	
SENS16	Mouth dryness	**0.52**	0.19	**0.28**		0.25	**0.58**	0.21	
SENS17	Mouth soreness	***0.61***	**0.28**	0.25		0.22	**0.39**	**0.28**	
SENS18	Hoarseness	***0.63***	0.13	0.23		0.08	**0.82**	0.15	
SENS19	Cracking voice	***0.68***	0.24	0.22		0.16	**0.77**	− 0.05	
SENS20	Throat pain	**0.57**	0.26	− 0.25		**0.34**	**0.30**	**0.48**	
SENS21	Throat feeling constricted	***0.61***	**0.30**	− **0.41**		0.19	0.10	**0.85**	
SENS22	Choking easily	**0.57**	**0.37**	− **0.44**		− 0.01	0.04	**0.82**	
SENS23	Difficulties swallowing	***0.66***	**0.34**	− 0.33		0.15	0.02	**0.84**	
SENS24	Throat problems limiting social life	***0.62***	**0.39**	− 0.18		**0.35**	0.16	**0.74**	
SENS25	*Problems from sensory organs affecting QoL*	***0.80***	0.11	− 0.13		***0.83***	0.06	0.11	
SENS26	Worrying about your sensory organs	***0.63***	0.16	0.14		***0.77***	0.03	0.20	
	Reliability	0.87	0.85	–*		0.83	0.83	0.86	

	Gastrointestinal functions	Dim 1	Dim 2			Dim 1	Dim 2		
GI1	Constipation	**0.36**	**0.83**			− 0.04	**0.44**		
GI2	Diarrhoea	**0.44**	− **0.67**			**0.53**	0.12		
GI3	Bowel urgency	**0.59**	− **0.50**			**0.81**	− 0.01		
GI4	Bowel leakage	***0.70***	− 0.09			**0.58**	0.10		
GI5	Bowel problems limiting social life	***0.76***	0.06			− 0.01	***0.60***		
GI6	*Bowel problems affecting QoL*	***0.87***	0.11			0.23	***0.60***		
GI7	Worrying about bowel problems	***0.84***	0.11			0.00	***0.96***		
	Reliability	0.86	0.80*			− *	0.87		

	Urinary tract functions	Dim 1	Dim 2			Dim 1	Dim 2		
UT1	Difficulties feeling the need to urinate	***0.63***	− **0.23**			0.16	**0.37**		
UT2	Difficulties emptying the bladder	**0.41**	0.08			− 0.03	**0.57**		
UT3	Night-time emptying of bladder	0.08	0.35			− 0.06	**0.41**		
UT4	Urinary urgency	0.00	**0.95**			− 0.02	***0.65***		
UT5	Stress incontinence	**0.34**	**0.38**			0.08	**0.30**		
UT6	Urinary problems limiting social activities	***0.66***	− 0.04			**0.28**	0.00		
UT7	*Urinary problems affecting QoL*	***0.77***	0.07			0.01	***0.78***		
UT8	Worrying about urinary problems	***0.86***	0.00			0.01	**0.58**		
	Reliability	0.82	–*			–*	0.71*		

	Work life	Dim 1	Dim 2			Dim 1	Dim 2		
WORK1	Health reasons for stopping working	0.18	**0.38**			**0.40**	**0.30**		
WORK2	Self-assessed capacity to work	− **0.86**	− 0.11			− **0.90**	0.04		
WORK3	Considering your health, still at present work in 2-year time	− **0.87**	0.05			− **0.74**	− 0.01		
WORK4	Work ability and physical demands	**0.89**	0.02			**0.92**	0.02		
WORK5	Work ability and mental / psychological demands	**0.87**	− 0.01			**0.77**	− 0.02		
WORK6	*Work problems affecting QoL*	0.09	***0.68***			**0.38**	**0.54**		
WORK7	*Financial problems affecting QoL*	0.17	***0.65***			**0.26**	**0.54**		
WORK8	Worry about future working life	− **0.21**	***0.93***			− 0.11	**0.88**		
WORK9	Worry about future work capacity	0.00	***0.83***			0.17	**0.71**		
WORK10	Worry about future finances	0.00	***0.83***			− 0.01	**0.68**		
	Reliability	0.91	0.93			0.91	0.88		

Italic values: Issue correlating to domain-specific QoL question

Underline values: Issue with strong loading but without correlation to domain-specific QoL question

^*^Cronbach's alpha, due to not enough factor loadings for omega coefficient to be calculated

^**^Omega coefficient = 0.80 without this question

BIC indicated a two-dimension solution for the fatigue domain (17,218.51; Additional file 1: Table S1), while AIC indicated a three-dimension solution (16,606.36). Reliability was good (0.84) to excellent (0.92) in the ICU survivor group and excellent (0.96) in the control group.

Compared to the non-ICU-treated control group, fewer issues were correlated with QoL in the ICU survivor group. While in the non-ICU-treated control group almost all issues correlated to QoL, in the ICU survivor group, only issues related to an effect on social life (*Tiredness affecting work*; *Tiredness affecting social activities*) correlated with QoL, thus focusing on a higher-level effect of fatigue more than the actual day-to-day symptoms as in the non-ICU-treated control group. Two issues, *Tired from reading* and *Tired from watching TV,* constituted a second dimension in the ICU survivor group, while these issues were part of the QoL dimension in the non-ICU-treated control group.

### Physical health

BIC indicated a four-dimension solution for the physical health domain (37,150.16; Additional file 1: Table S1), while AIC indicated a six-dimension solution (35,290.18). Reliability was good in both groups (0.85 and 0.89 in the ICU survivor group and 0.82 and 0.89 in the control group).

No major differences in dimensionality were seen between the two groups, including very few correlations with QoL. For both groups, *General physical health* and *Future worries regarding physical health* correlated to QoL, while the ICU survivor group also correlated social limitations of physical health to QoL.

### Pain

A four-dimension solution was retrieved for the pain domain (BIC 29949.91; AIC 29005.65; Additional file 1: Table S1). Reliability was good (0.81 and 0.87) in the ICU survivor group and questionable (0.69) to good (0.82) in the control group.

*Pain stopping planned activity* affected QoL in both groups and was the only issue related to QoL in the non-ICU-treated control group. While the ICU survivor group correlated *Worrying about pain* to QoL, this was simply an issue for the non-ICU-treated control group without an effect on QoL. Both groups shared a dimension with issues related to pain and sleep (*Painkillers for sufficient sleep*; *Pain makes going to sleep difficult*), although the ICU survivor group also included the issues of *Painkillers to manage ADL* in the same dimension.

### Mental health

A three-dimension solution was retrieved for the mental health domain (BIC 28624.14; AIC 27776.05; Additional file 1: Table S1). Reliability was good to excellent (0.83 and 0.94) in the ICU survivor group and acceptable to good (0.79 and 0.85) in the control group.

Large differences were seen between the two groups regarding Mental health issues and QoL. Most notably, while the ICU survivor group correlated multiple issues associated with a low mood state to QoL (*Feeling low-spirited*; *Feeling depressed*; *Periods of anxiety*; *Feelings of hopelessness*; *Feeling of life being meaningless*; *Low self-confidence*; *Low self-esteem*), no issues correlated to QoL in the control group. The groups showed almost identical patterns in the other two dimensions: one containing *Feeling short-tempered* and *Losing patience easily*, and one containing the reversely coded *Able to laugh at things* and *Able to look forward to things*.

### ADL

A three-dimension solution was retrieved for the ADL domain (BIC 13808.47; 13,257.21; Additional file 1: Table S1). Reliability was good to excellent (0.85 and 0.92) in the ICU survivor group and excellent (0.94 and 0.96) in the control group.

Several issues correlated with QoL in the ICU survivor group: *Avoiding taking public transport*, activities related to managing a household (*Help shopping*; *Help cooking*; *Help with housework*), and *Worrying about daily activities*. The dimension unrelated to QoL in the ICU survivor group contained more intimate issues (*Help showering*; *Help getting dressed*; *Help visiting the toilet)*. Compared with the ICU survivor group, no issues in the non-ICU-treated control group correlated with QoL, although most issues had a strong loading.

### Sleep

A three-dimension solution was retrieved for the sleep domain (BIC 14488.26; AIC 14034.50; Additional file 1: Table S1). Reliability was acceptable (0.73) to good (0.81) in the ICU survivor group and questionable (0.63) to acceptable (0.79) in the control group.

For the ICU survivor group, the only issue related to QoL was *Worries about sleep*. In the control group, no issue was related to QoL, and *Worries about sleep* was instead correlated to *Need for sleeping pills*.

### Appetite & alcohol use

A three-dimension solution was retrieved for the appetite and alcohol domain (BIC 13473.31; AIC 13018.84; Additional file 1: Table S1). In the ICU survivor group, reliability varied from acceptable (0.75) to good (0.86), while in the control group, it varied between good (0.85) and excellent (0.95).

The groups were essentially similar in regard to QoL, with *Worries about appetite* and *Worries about alcohol* constituting the QoL dimension in both groups. In addition, only the ICU survivor group included *Poor appetite* in the appetite QoL dimension.

### Sexual health

A two-dimension solution was retrieved for the Sexual health domain (BIC 14029.44; AIC 13655.72; Additional file 1: Table S1). Reliability was questionable (0.67) to excellent (0.90) in the ICU survivor group and good (0.82) to excellent (0.90) in the non-ICU-treated control group.

The two groups were similar across all dimensions in the Sexual health domain apart from *Surgical scars affecting sex life* that was related to QoL in the ICU survivor group and not in the non-ICU-treated control group. While *Worries about sex life* was related to QoL in both groups, a dimension including *Sex drive*, *Sexual activity*, *Sex life,* and *Orgasm* was not related to QoL in both groups.

### Sensory functions

A three-dimension solution was retrieved for the Sensory functions’ domain (BIC 24696.47; AIC 23869.07; Additional file 1: Table S1). Reliability was good (0.85–0.87) in the ICU survivor and control groups (0.83–0.86).

Several differences could be seen between the groups. First, while the non-ICU-treated control group related several sound- and hearing-related issues to QoL, the ICU survivor group only related *Reduced hearing limiting social life* to QoL. Instead, issues related to voice and throat problems (*Hoarseness*; *Cracking voice*; *Throat feeling constricted*; *Difficulties swallowing; Throat problems limiting social life*), as well as *Reduced taste* and *Reduced smell,* correlated to QoL in the ICU survivor group.

### Gastrointestinal functions

A two-dimension solution was retrieved for the gastrointestinal domain (BIC 8918.46; AIC 8451.03; Additional file 1: Table S1). Reliability was good in both groups: 0.80 and 0.86 in the ICU survivor group and 0.87 in the non-ICU-treated control group.

While both groups correlated *Bowel problems limiting social life* and *Worrying about bowel problems* to QoL, the remaining issues differed. The ICU survivor group also correlated *Bowel leakage* to QoL, while no other issues correlated to QoL in the non-ICU-treated control group.

### Urinary tract functions

A two-dimension solution was retrieved for the Urinary tract functions domain (BIC 9467.89; AIC 9197.17; Additional file 1: Table S1). Reliability was good (0.82) in the ICU survivor group and acceptable (0.71) in non-ICU treated groups.

Differences were seen between the two groups regarding dimensionality. While *Urinary urgency* was an issue for both groups, only the non-ICU-treated control group correlated it to QoL. Instead, the ICU survivor group related socially limiting issues to QoL (*Difficulties feeling the need to urinate*; *Urinary problems limiting social activities*).

### Work life

A two-dimension solution was retrieved for the Work life domain (BIC 10313.21; AIC 9973.57; Additional file 1: Table S1). Reliability was excellent (0.91 and 0.93) in the ICU survivor group and good (0.88) to excellent (0.91) in the control group.

While the dimensionality of Work life is similar between the two groups, the relationship to QoL differs. Both groups loaded questions about worries (Future working life; Future work capacity; Future finances) in the same dimension, but while the ICU survivor group related these ussies to QoL, the non-ICU-treated control group did not. Both groups loaded issues regarding work capacity (*Self-assessed capacity to work*; *Still at present work in 2 years*; *Work ability and physical/mental demands*) into the same dimension.

### Cognition

A three-dimension solution was retrieved for the cognitive domain (BIC 39713.05; AIC 38384.60; Additional file 1: Table S1). Reliability was good (0.84) to excellent (0.90) in the ICU survivor group and good (0.85–0.87) in the control group.

No dimension correlated to QoL in either of the groups. The groups were somewhat similar in the dimensionality regarding language (*Difficulties finishing sentences*; *Losing the thread easily* shared by both groups). However, while the ICU survivor group separated issues mixing language and memory (*Don't remember what you said*; *Don't remember what you have done*; *Think you have done something but you haven't*) into a second dimension, these issues were still part of the language dimension in the non-ICU-treated control group, while more purely memory-related issues constituted the second dimension of this group (*Need for memos*; *Difficulties remembering names/general knowledge/what you have read/previous TV episodes*). In relation to the non-ICU-treated control group, the ICU survivor group had a dimension dominated by issues regarding executive abilities (*Difficulties taking initiative*; *Difficulties prioritising*; *Difficulties concentrating*; *Difficulties finding alternative solutions*).

## Discussion

In this cross-sectional study on burdens after intensive care, we explored factorial patterns focusing on quality of life in 218 different issues in 13 domains and compared results between adult ICU survivors and a randomised non-ICU-treated control group, matched for age and sex. Our findings show large variations over the different domains evaluated. For example, while an effect on QoL correlated to 43% of Mental health issues and 33% of ADL issues in the ICU survivor group, it did not correlate with any issues in these domains for the non-ICU-treated control group. Contrary to this, the patterns were almost identical between the two groups regarding Physical health and Sexual health.

Furthermore, our study shows that about one-fourth of the issues experienced by ICU survivors and about one-tenth of the issues experienced by controls are related to QoL. These findings illustrate the potential problem with weighting different domains of QoL equally. Nakamura et al. examined data on over 13,000 elderly patients participating in the American prospective Health and Retirement Study and found that psychological outcomes were substantially more important for health and well-being than for example personal finance [21]. In another study, Hsieh examined the plausibility of an equal-weight, equal-importance approach in well-being research [22]. Analysing two different datasets on life satisfaction in general, a large American longitudinal household survey (*n* = 5049) and an online Chinese survey (*n* = 1620), he found a significant difference in the importance of different domains in life in both datasets. Our findings are in line with both of these studies.

Other differences between the ICU survivor group and the non-ICU-treated control group in our study should be noted. Issues related to a more general social perspective are somewhat more prone to correlate to QoL in ICU survivors than in non-ICU-treated controls: For example, only the ICU survivor group related adverse social effects of throat problems and urinary tract problems to QoL, and in Fatigue the ICU survivor group only correlated the social aspects to QoL. In contrast, the non-ICU-treated control group correlated virtually all fatigue issues to QoL. The consequences of social isolation after intensive care have previously been studied. For example, Falvey et al. recently used data from a large national survey study to show an association between post-ICU isolation and both disability burden and mortality [23].

We found no correlation between cognitive dysfunction and QoL in either of the groups. This finding does not question the presence of cognitive dysfunction but rather suggests that it may not necessarily affect QoL. This finding may align with that of Nedergaard et al., who let ICU patients, ICU physicians, and ICU nurses rank the importance of different post-ICU outcomes [24]. They found that patients ranked cognitive dysfunction not only lower than what physicians and nurses did but also overall lower than areas such as physical and mental health, ADL, and fatigue. A possible explanation is that cognitive dysfunction decreases the ability to identify these problems correctly [25].

An additional finding is that while the dimensionality in the Work life domain is similar between the two groups, only the ICU survivor group correlates future worries regarding work life to QoL. Difficulties returning to work after intensive care, even long-term, have been previously well described. A systematic review and meta-analysis showed that one-third of previously employed ICU survivors were still unemployed five years after hospital discharge, plausibly describing the reality explaining the lack of hope in our study [26].

Fatigue after intensive care is extensively studied. Prevalence seems highly variable, with a recent systematic review showing a range from 13% to over 80% [27]. However, our study adds to this knowledge by relating findings in ICU survivors to those in a control group. We found that the ICU survivors only correlated the social effects of fatigue to QoL, while the non-ICU-treated control group correlated almost all effects of fatigue to QoL. This finding may illustrate the effect of coping in the ICU survivor group, where certain issues are learned to live with as existing symptoms and only specific aspects of them are felt affecting QoL. In line with this interpretation is the finding that only the non-ICU-treated control group correlated future worries regarding fatigue to QoL as opposed to the ICU survivor group.

While our study found differences in factor dimensionality between the ICU survivor group and the non-ICU-treated control group, there are similarities between the two groups as well, for example, in the domain of Physical health. This may partially be explained by the "disability paradox". In a recent survey by Iezzoni et al., over 80% of participating physicians assumed that people with significant disabilities have worse QoL than people without disabilities [28]. However, as shown by Albrecht and Devlieger, many with significant disability report having a good or excellent QoL [29]. This discrepancy between the perceptions of healthcare providers and patients has also been shown within intensive care, where Detsky et al. showed that the discriminative accuracy of ICU physicians to predict QoL 6 month after ICU was low (sensitivity 49%; specificity 51%) [30].

Our study is, to our knowledge, the first to differentiate the post-ICU issues affecting QoL from those that do not, giving a more detailed view of the burdens after intensive care than previously shown. Future questionnaires focusing on post-ICU trajectories may separate QoL-related issues from other day-to-day issues, improving granularity in outcomes from interventional trials. The finding that not all issues relate to QoL further adds to the question of who should measure QoL—patients themselves or clinicians and proxies. In their qualitative study on ICU survivors' problems, Nedergaard et al. found it necessary to have QoL as a separate issue instead of as a more general phenomenon since ICU survivors repeatedly brought QoL up as a separate problem [24].

We have chosen to study ICU survivors as a homogenous group, with the risk of not identifying potential subgroup-specific aspects related to admission diagnosis, age, or other variables. Although it is possible that specific subgroups would present alternative patterns, our questions were developed aiming for generalisability across a general intensive care population.

The lack of longitudinal design in our study precludes us from knowing whether differences in patterns between our two groups result from a response shift from a long-term adaptation after intensive care with new priorities. A future longitudinal study design with a pre-admission status would be better suited to characterise this change over time. For example, while initial physical health problems might be learned to cope with over time and show a decreasing association with QoL, persistent difficulties returning to work might, hypothetically, increasingly affect QoL.

Finally, this is an exploratory factor analysis. As such, it tests our previously found issues without an a priori hypothesis about the number of latent variables, aiming at determining the underlying structure. Future steps need to test the significance of these findings through confirmatory factor analysis and may, together with the current findings, be of aid in a reduction of the number of questions and the creation of a final questionnaire.

## Conclusions

In this study on burden after intensive care, we found that most issues experienced by ICU survivors are unrelated to quality of life. Our findings indicate that the consequences of post-ICU issues may play a more significant role in affecting QoL than the issues themselves; issues restricting and affecting social life and work life were more related to QoL in ICU survivors than in non-ICU-treated controls.

Caution is advised before associating all post-ICU problems with an effect on quality of life.

### Supplementary Information

**Additional file 1: Table S1.** Model fit indices and dimensionality solutions for all domains.

## Data Availability

The datasets generated during and/or analysed during the current study are not publicly available due to them containing information that could compromise research participants' privacy/consent, but they are available from the corresponding author upon reasonable request.

## Abbreviations

EFA: Exploratory factor analysis
ICU: Intensive care unit
MG-EFA: Multi-group exploratory factor analysis
QoL: Quality of life
